# The dual nature of mismatch repair as antimutator and mutator: for better or for worse

**DOI:** 10.3389/fgene.2014.00287

**Published:** 2014-08-21

**Authors:** Sara Thornby Bak, Despoina Sakellariou, Javier Pena-Diaz

**Affiliations:** Department of Neuroscience and Pharmacology and Center for Healthy Aging, University of CopenhagenCopenhagen, Denmark

**Keywords:** non-canonical mismatch repair, antibody diversification, class switch recombination, somatic hypermutation, neurodegenerative diseases, trinucleotide repeats, chromatin modifiers

## Abstract

DNA is constantly under attack by a number of both exogenous and endogenous agents that challenge its integrity. Among the mechanisms that have evolved to counteract this deleterious action, mismatch repair (MMR) has specialized in removing DNA biosynthetic errors that occur when replicating the genome. Malfunction or inactivation of this system results in an increase in spontaneous mutability and a strong predisposition to tumor development. Besides this key corrective role, MMR proteins are involved in other pathways of DNA metabolism such as mitotic and meiotic recombination and processing of oxidative damage. Surprisingly, MMR is also required for certain mutagenic processes. The mutagenic MMR has beneficial consequences contributing to the generation of a vast repertoire of antibodies through class switch recombination and somatic hypermutation processes. However, this non-canonical mutagenic MMR also has detrimental effects; it promotes repeat expansions associated with neuromuscular and neurodegenerative diseases and may contribute to cancer/disease-related aberrant mutations and translocations. The reaction responsible for replication error correction has been the most thoroughly studied and it is the subject to numerous reviews. This review describes briefly the biochemistry of MMR and focuses primarily on the non-canonical MMR activities described in mammals as well as emerging research implicating interplay of MMR and chromatin.

## INTRODUCTION

The mismatch repair (MMR) system provides two main genetic stabilization functions; it is involved in the correction of errors generated during replication that escape polymerase proofreading and ensures the fidelity of recombination. Such a corrective role was first proposed to explain gene conversion in fungi (Holliday, 1974). Studies using bacteria and yeast uncovered MMR as a long patch correction system and identified its protein components (Grilley et al., 1990). The MMR process was then reconstituted using bacterial (Lahue et al., 1989), yeast (Bowen et al., 2013), and mammalian proteins (Constantin et al., 2005; Zhang et al., 2005). Defects in this pathway were shown to give rise to a mutator phenotype in bacteria and yeast with characteristic traits at repetitive sequences of simple nature, microsatellites (microsatellite instability, MSI; Levinson and Gutman, 1987; Strand et al., 1993). The observation that a subset of colorectal tumors contain a large number of mutations in microsatellite sequences was subsequently explained by the finding that these tumors were defective in MMR (Fishel et al., 1993; Leach et al., 1993; Jiricny, 1994; Modrich and Lahue, 1996). The discovery that MMR defects predispose to cancer (Lynch syndrome) highlighted the relevance of MMR in human disease and renewed the interest in MMR proteins, their structure, mechanisms of action and gene variants that may contribute to the disease (Boland and Goel, 2010). The mechanistic insights obtained by these studies did advance our understanding on how hereditary sequence variants in the minimal human MMR system affect the MMR function and hence predispose to the DNA instabilities linked to cancer predisposition. The list of cancer types where MMR malfunction has been observed expanded to include the most frequent hereditary predisposition to colorectal cancer along with increased risk for development of endometrial, ovarian, gastric, small bowel, urothelial, brain, hepatobiliary, pancreatic, bladder, kidney, prostate and breast cancers, and hematological malignances (Scott et al., 2001; Umar et al., 2004; Grindedal et al., 2009; van Oers et al., 2010; Wimmer and Kratz, 2010; Buerki et al., 2012; Win et al., 2012a,b; Vasen et al., 2013). The ability to predict cancer predisposition by analyzing the sequence variants for the MMR genes also contributed to better management of patients and their relatives and resulted in reduced mortality (Jarvinen et al., 2009). Therefore, the characterization of such gene variants has become of prime interest and is nowadays a multidisciplinary international endeavor (Thompson et al., 2014). The efforts made in understanding MMR mechanism and function also led to the discovery of new roles for MMR. MMR was found to be involved in DNA damage signaling and intriguingly also in mutagenic processes such as somatic hypermutation (SHM), class switch recombination (CSR), and instability of trinucleotide repeats (TNRs; Hsieh, 2001; Li, 2008; Pena-Diaz and Jiricny, 2012; Edelbrock et al., 2013; Jiricny, 2013). This review describes first the components of mammalian MMR and their mode of action and then focuses on DNA transactions in which MMR contradicts its role as antimutator to become a mutator.

## THE BIOCHEMISTRY OF MAMMALIAN MMR

**FIGURE 1:**
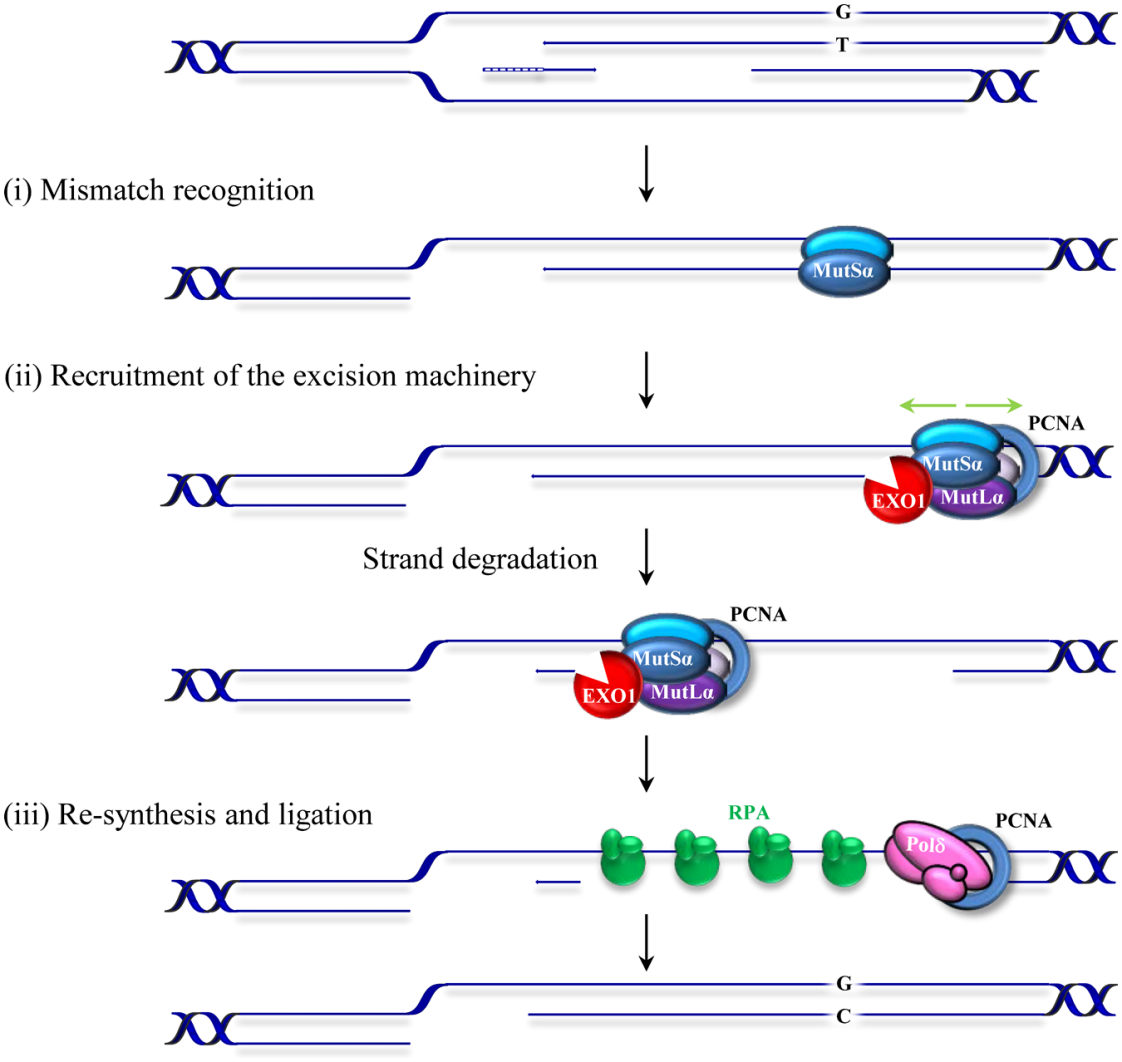
**Schematic representation of postreplicative mismatch repair in human cells.** The canonical MMR process commences by the binding of the MSH2/MSH6 heterodimer, MutSα, to a mismatch (in this figure a G/T mismatch in the leading strand resulting from misincorporation during replication of thymidine opposite to guanosine). Upon binding, MutSα undergoes an ATP-driven conformational change and recruits the MLHl/PMS2 heterodimer (MutLα). This complex can translocate in either direction along the DNA contour (green arrows). When it encounters a strand discontinuity (such as a gap between Okazaki fragments in the lagging strand or a PMS2 induced nick in the leading strand, not shown) PCNA binding (blue circle) and loading of an exonuclease (EXO1) initiate degradation of the nicked strand that will terminate past the mismatch. The resulting RPA-stabilized single-stranded gap is then filled in by the replicative polymerase and the remaining nick sealed by DNA ligase I. Small insertion/deletion loops (not shown) are corrected in a similar fashion by a MutSβ (MSH2/MSH3) initiated process.

Replication errors represent a considerable threat to genomic integrity. Failure to repair base–base mismatches and insertion/deletion loops (IDLs) arising during DNA replication increases mutation frequencies by two to three orders of magnitude. MMR associates with replication factories (Hombauer et al., 2011; Lopez-Contreras et al., 2013; Sirbu et al., 2013) and targets the newly synthesized DNA strand for repair thereby contributing to the fidelity of replication. MMR achieves this feat by a sequential mechanism comprising mismatch recognition, excision, and resynthesis steps. This process has been described in detail in several reviews (Kunkel and Erie, 2005; Jiricny, 2006; Modrich, 2006; Hsieh and Yamane, 2008). Briefly, the reaction commences by the binding of the MutS heterodimer to a mismatch (**Figure 1**). The MutS heterodimer is formed by either MSH2/MSH6 (MutSα) or MSH2/MSH3 (MutSβ). Two other homologs, MSH4 and MSH5, have specific roles in meiosis and have been discussed previously (Snowden et al., 2004; Her et al., 2007). The MutSα complex recognizes single base mismatches and 1–2 nucleotide IDLs, while the MutSβ complex recognizes larger loops. The mechanisms of lesion recognition by MutSα and MutSβ differ but in both cases binding leads to bending of DNA (Warren et al., 2007; Gupta et al., 2012). MutS heterodimers belong to the ABC transporter superfamily and contain ATP binding domains essential for MMR. Following substrate recognition, MutS undergoes an ADP–ATP exchange-driven conformational change into a sliding clamp and recruits the MutL heterodimer. There are several MutL homologs; MutLα, MutLβ, and MutLγ that belong to the GHKL ATPase family (Dutta and Inouye, 2000). MutLα (MLH1/PMS2 heterodimer) is the prevalent homolog in MMR. MutLβ (MLH1/PMS1) appears to lack a function in MMR, whereas MutLγ (MLH1/MLH3) contributes to some extend to MMR *in vitro* (Cannavo et al., 2005) but is primarily involved in meiotic recombination (Lipkin et al., 2002). The complex formed by MutS–MutL can translocate in either direction along the DNA contour in search of a strand discontinuity. When it encounters a strand discontinuity (such as a gap between Okazaki fragments) bound by PCNA, loading of the exonuclease EXO1 initiates degradation of the nicked strand that will terminate past the mismatch. Additionally, the latent endonuclease activity harbored by MutLα (Kadyrov et al., 2006) may provide an entry site for EXO1-dependent excision or for polymerase-dependent strand displacement reactions (Kadyrov et al., 2009). The resulting single-stranded gap is stabilized by RPA and then filled in by polymerase δ. The remaining nick is sealed by DNA ligase I. The physical interactions of MutS and MutL with the replication factor PCNA and the constitutive presence of the MMR machinery at replication factories support the role of MMR as a postreplicative repair mechanism. However, several studies indicate that MMR proteins may also function outside of S-phase (Brooks et al., 1996; Zlatanou et al., 2011; Pena-Diaz et al., 2012). In contrast to the classical MMR activity described above, some of the activities derived from this replication-uncoupled MMR are mutagenic. Such a mutagenic non-canonical MMR (ncMMR) has been found to influence immunoglobulin diversification and the stability of TNRs.

## MMR IN IMMUNOGLOBULIN DIVERSIFICATION

### GENERATION OF ANTIBODY DIVERSIFICATION IN HUMANS

**FIGURE 2:**
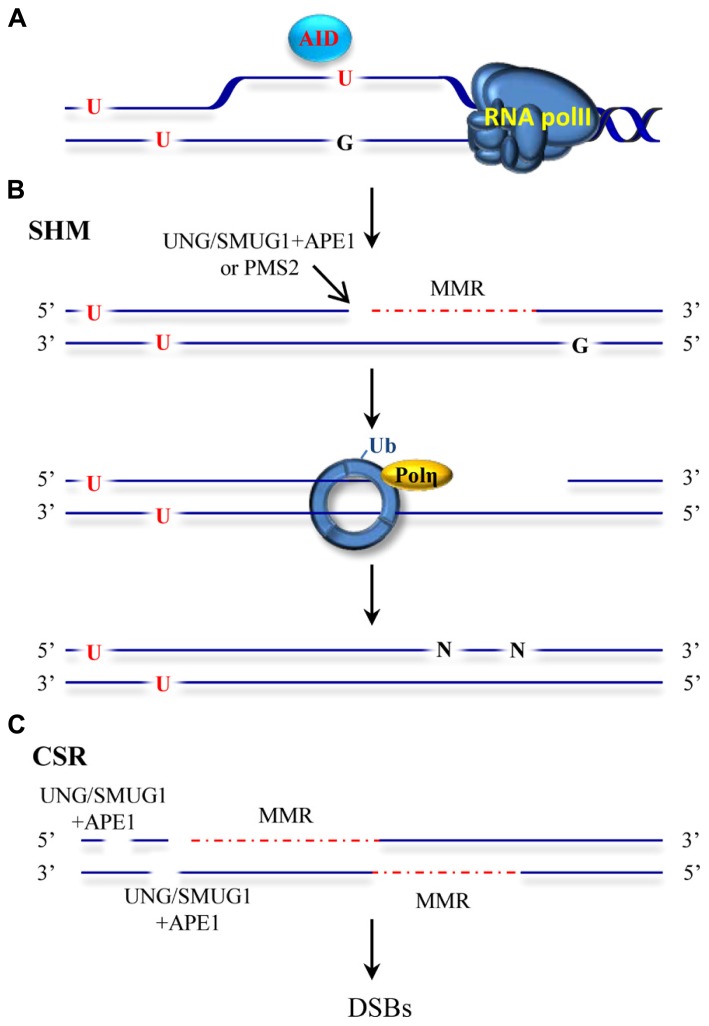
**ncMMR as a mediator in SHM and CSR. (A)** AID deaminates cytosine to uracil in single-stranded DNA such as in DNA that is being transcribed. U:G mismatches can be recognized by the BER and MMR machineries. **(B)** An inefficient BER can lead to excision of the uracil by UNG or SMUG1 glycosylases and to an incision by APE1. MMR loaded at a different mismatch, can use this APE incision as an entry point for EXO1-mediated degradation. Alternatively, PMS2 endonuclease can generate the required entry site. The single-stranded DNA generated by EXO1 is not readily filled and promotes PCNA-Ub and recruitment of Pol η. Resynthesis by the error prone Pol η leads to mutations at different sites than the original deaminated cytosine. **(C)** Incisions generated by BER and/or MMR-dependent strand degradation can lead to DSBs when the degradation tracks and breaks are in close proximity on opposite strands. DSBs induction initiates recombination events during CSR. The red dashed line indicates MMR-dependent strand degradation.

Our immune system is able to generate a staggering repertoire of antibodies in order to deal with the variety of antigens that we may encounter during our life time. The information required to synthesize this large number of antibodies is not directly contained in our limited genome. Instead, several mutagenic processes taking place at the immunoglobulin locus are responsible for altering the genetic information to create sufficient diversity. Antibody diversity is generated in a two-stage process. Early in B cell development, DNA breakage and rejoining events between variable (V), diversity (D) and joining (J) gene segments assemble immunoglobulin genes and allow the production of a primary repertoire of low affinity IgM antibodies (Jung et al., 2006; Schatz and Swanson, 2011). In mammals, a second diversification process that alters the sequence and structure at the immunoglobulin genes occurs after exposure of a B cell to an antigen. This secondary process entails SHM and CSR mechanisms and generates different classes of antibodies with higher affinities and specificities (Maizels, 2005; Di Noia and Neuberger, 2007; Teng and Papavasiliou, 2007; Peled et al., 2008; Stavnezer et al., 2008). SHM introduces mutations in the variable region of the Ig gene while CSR recombines the variable region to a downstream constant region in the Ig locus by a double-strand break (DSB) induced event. SHM and CSR are initiated by a shared event involving targeted DNA deamination catalyzed by the enzyme activation-induced deaminase (AID; Muramatsu et al., 1999, 2000; Bransteitter et al., 2003; Chaudhuri et al., 2003; Dickerson et al., 2003). The discovery of AID represented a milestone in the immunology field and initiated further studies into the molecular basis of SHM and CSR processes (Delker et al., 2009). AID converts cytosines to uracils in single-stranded DNA (Bransteitter et al., 2003; Chaudhuri et al., 2003; Dickerson et al., 2003; **Figure 2A**) and initiates mutagenic processes with the participation of low fidelity DNA polymerases and DNA repair pathways including base excision repair (BER), MMR, classical non-homologous end-joining and alternative end-joining. Ample genetic evidence has substantiated the seemingly paradoxical involvement of BER and MMR in this mutagenic process. Moreover, mutations in MMR proteins that affect different catalytic functions or physical interactions with other components of this pathway have been shown to affect immunoglobulin diversification processes (Chahwan et al., 2011). This review summarizes the current mechanistic model proposed for mutagenic MMR.

### MMR AS A MUTATOR AT THE IMMUNOGLOBULIN LOCUS

How is ncMMR engaged at the immunoglobulin locus? AID-mediated cytosine deamination results in a U/G mismatch in the DNA that leads to several outcomes. During SHM, if the mismatch is not corrected, replication across U will lead to C/G to T/A transitions. A second type of mutations is dependent on error-prone BER. BER initiated predominantly by the uracil–DNA glycosylase UNG, or to a minor extent by the backup activity of SMUG1 (Dingler et al., 2014) may correct the mismatch and restore the original sequence or, when incomplete, leave abasic sites that are mis-repaired by error-prone polymerases. These events take place at the deaminated cytosine site and leads to both transitions and transversions (Sousa et al., 2007). These activities were confirmed by the finding that ablation of UNG in mice leads to accumulation of uracil in the DNA of immunoglobulin genes, and to a significant increase in transition mutations at C/G pairs (Rada et al., 2002; Maul et al., 2011). A third type of frequent mutations occurring at A/T base pairs and therefore not affected directly by the deamination of cytosine were shown to arise by a different mechanism (**Figure 2B**). This third mode of addressing uracil in DNA required long path DNA repair processes with a propensity to introduce errors. Genetic evidence suggested the involvement of MMR proteins, EXO1, mono-ubiquitylation of PCNA (PCNA-Ub) and primarily the translesion synthesis polymerase η (Bardwell et al., 2004; Delbos et al., 2007; Krijger et al., 2009; Chahwan et al., 2012a; Saribasak and Gearhart, 2012). Upon MutSα recognition of the U/G mismatch the complex slides along DNA in search of an entry site for EXO1 loading, and once such an entry site is found, initiates strand degradation. The gaps formed in this process are believed to persist and to trigger PCNA-Ub and recruitment of pol η. In absence of MSH2, mutations at A:T sites are drastically reduced but not completely abolished. In this scenario, BER is suggested to provide a backup role for the recruitment of pol η during SHM (Delbos et al., 2007). Two major open questions about this process remain: (i) which enzymatic activity generates the entry site for EXO1-dependent degradation and (ii) what distinguishes high fidelity from error prone MMR. (i) The answer to the first question may lie in the potential of AID to create clustered mutations at the Ig locus (Storb et al., 2009). In this scenario, BER may introduce a strand discontinuity that can be used by proximally loaded MMR proteins as entry point for EXO1 (Schanz et al., 2009). A complementary model substantiated by recent findings suggests that in absence of proximal entry sites, a cryptic endonuclease activity harbored by PMS2 may serve as a back-up for the DNA incision required to initiate EXO1-dependent strand degradation (Pluciennik et al., 2010; Pena-Diaz et al., 2012; Zivojnovic et al., 2014). Overlapping roles of BER and ncMMR have been demonstrated and endorse these two possibilities (Rada et al., 2004; Shen et al., 2006). Whereas strand discontinuities created by BER may serve to direct MMR to the same strand containing the nick, in absence of entry sites, the back-up cleavage by PMS2 endonuclease is largely without strand bias (Pluciennik et al., 2010; Pena-Diaz et al., 2012). The interplay between BER and MMR thus may affect the strand bias observed for mutations at A/T sites (preferential targeting of A nucleotides for mutation within WA motifs on the non-transcribed strand). The source of the strand bias observed at A/T sites though remain controversial (Franklin and Blanden, 2008; Frieder et al., 2009; Steele, 2009; Roa et al., 2010). (ii) Once EXO1 is loaded and strand degradation takes place, what distinguishes high-fidelity from error-prone MMR? Whereas high-fidelity MMR is coupled to replication, ncMMR acting in SHM and CSR processes may take place outside of S-phase. The mutagenic ncMMR thus may function in an environment where replicative polymerases are scarce and dNTP pools suboptimal. This could lead to inefficient refilling of the single-stranded gaps formed during the repair process, which would in turn elicit PCNA-Ub and promote refilling of the gap by error-prone polymerases. In this model, DNA lesions addressed by MMR outside S-phase promote MMR-dependent PCNA-Ub. This is supported by several studies showing that oxidative and alkylating DNA damage can elicit MMR-dependent PCNA-Ub independently of the cell-cycle phase (Schroering and Williams, 2008; Zlatanou et al., 2011; Pena-Diaz et al., 2012).

CSR similarly to SHM requires AID, BER, and MMR proteins. CSR requires the formation of DSBs in highly repetitive switch regions located upstream of each of the heavy chain constant region genes (**Figure 2C**). These breaks are subsequently processed by canonical non-homologous end-joining (C-NHEJ) that seals DNA ends with little or no homology or by alternative end-joining (A-EJ) that requires microhomology for ligation (Boboila et al., 2012; Cortizas et al., 2013). How these DSBs are created is not entirely clear. BER may create single strand-breaks on opposite strands that when sufficiently close lead to DSBs (Masani et al., 2013). Fortuitous overlap of MMR-generated gaps with BER breaks or other MMR-induced gaps in the opposite strand provides an additional explanation for the formation of DSBs (Peron et al., 2008; van Oers et al., 2010). Strikingly, while SHM is largely independent of MutLα, the formation of DSBs during CSR requires the PMS2 endonuclease activity (van Oers et al., 2010). MMR can be initiated using strand discontinuities provided by BER and therefore does not strictly require PMS2 endonuclease activity (Genschel and Modrich, 2003). In this scenario, the gaps formed by MMR are in the same strand than the original strand discontinuity provided by BER. On the other hand, formation of gaps on the opposite strand of nicks generated by BER is aided by the lack of strand bias exhibited by MMR in absence of nearby nicks (Pluciennik et al., 2010; Pena-Diaz et al., 2012). This therefore increases the likelihood of DSB formation and it may partly explain the critical requirement of PMS2 endonuclease activity during CSR. MMR proteins may have additional functions beyond their major role converting AID DNA damage into suitable broken ends for C-NHEJ and A-EJ pathways. Recent studies suggest that MMR proteins may influence the pathway choice for resolution of the DSBs formed during CSR (Eccleston et al., 2011; Chahwan et al., 2012b; Cortizas et al., 2013). Biochemical evidence substantiating the models for DSBs formation during CSR and the potential role of MMR proteins in pathway choice for DSBs resolution is still missing.

Currently, it is not known whether the ncMMR mutagenic activity is engaged exclusively at AID deaminated sites in the immunoglobulin locus. AID may act on many non-Ig genes (Liu et al., 2008; Chiarle et al., 2011; Klein et al., 2011; Staszewski et al., 2011; Fear, 2013) and spontaneous deamination of cytosine to uracil is also a frequent event (∼200 per mammalian genome per day; Kavli et al., 2007). Therefore, lesions that can be recognized by MMR are not locus specific and MMR mutagenic activities may be more frequent than anticipated. The interplay or competition between BER and MMR activities, the regulation of the access of error-prone polymerases and the timing of repair related to the cell cycle are likely to influence the balance between high-fidelity and error-prone DNA repair in these loci.

## MMR IN NEURODEGENERATIVE DISEASE

### REPEAT INSTABILITY AS A CAUSE OF HUMAN DISEASE

Expansion of simple repeats in genomic DNA is the underlying cause of over 30 human neurodegenerative and neuromuscular inherited diseases such as Huntington’s disease (HD), myotonic dystrophy type 1 (DM1), fragile X syndrome type A (FRAXA), Friedreich’s ataxia (FRDA), and spinocerebellar ataxias (SCAs). Unstable repeats are polymorphic and show a normal range in healthy individuals, and a pathological range, i.e., above a threshold length, associated with clinical manifestations. Instability can occur during both meiotic and mitotic divisions and at various stages of the cell cycle (Nag, 2003; McMurray, 2010). Several of the repeat expansion-associated diseases show anticipation, in which subsequent generations display earlier disease onsets. Otherwise, somatic instability accounts for increases with age towards larger size of the repeats in a tissue-dependent manner correlating with progression of the symptoms. Long repeats exceeding a determined threshold tend to be more unstable and both gametic and mitotic instability becomes more likely with increasing repeat length. The unstable repeats can be found at different regions of their resident genes (Mirkin, 2007) and the etiology of the diseases caused by their expansion reflects this diversity. Repeat expansions can cause disease by a variety of both loss and gain of function pathways, interfering with the expression or properties of the gene products, affecting splicing or antisense regulation. The most common unstable disease-associated DNA repeats are TNRs including CAG, CTG, CGG, and GAA triplets and their expansion is thought to be linked to their ability to form unusual secondary structures (Gacy et al., 1995; Mirkin, 2007). Several mechanisms including errors during DNA replication, meiotic recombination, transcription, DNA repair, and chromatin remodeling have been proposed to contribute to the observed instability (Lin et al., 2009; Lopez Castel et al., 2010; McMurray, 2010; Kim and Mirkin, 2013), but their relative contribution remains unknown.

### MMR AS A SOURCE OF REPEAT INSTABILITY

**FIGURE 3:**
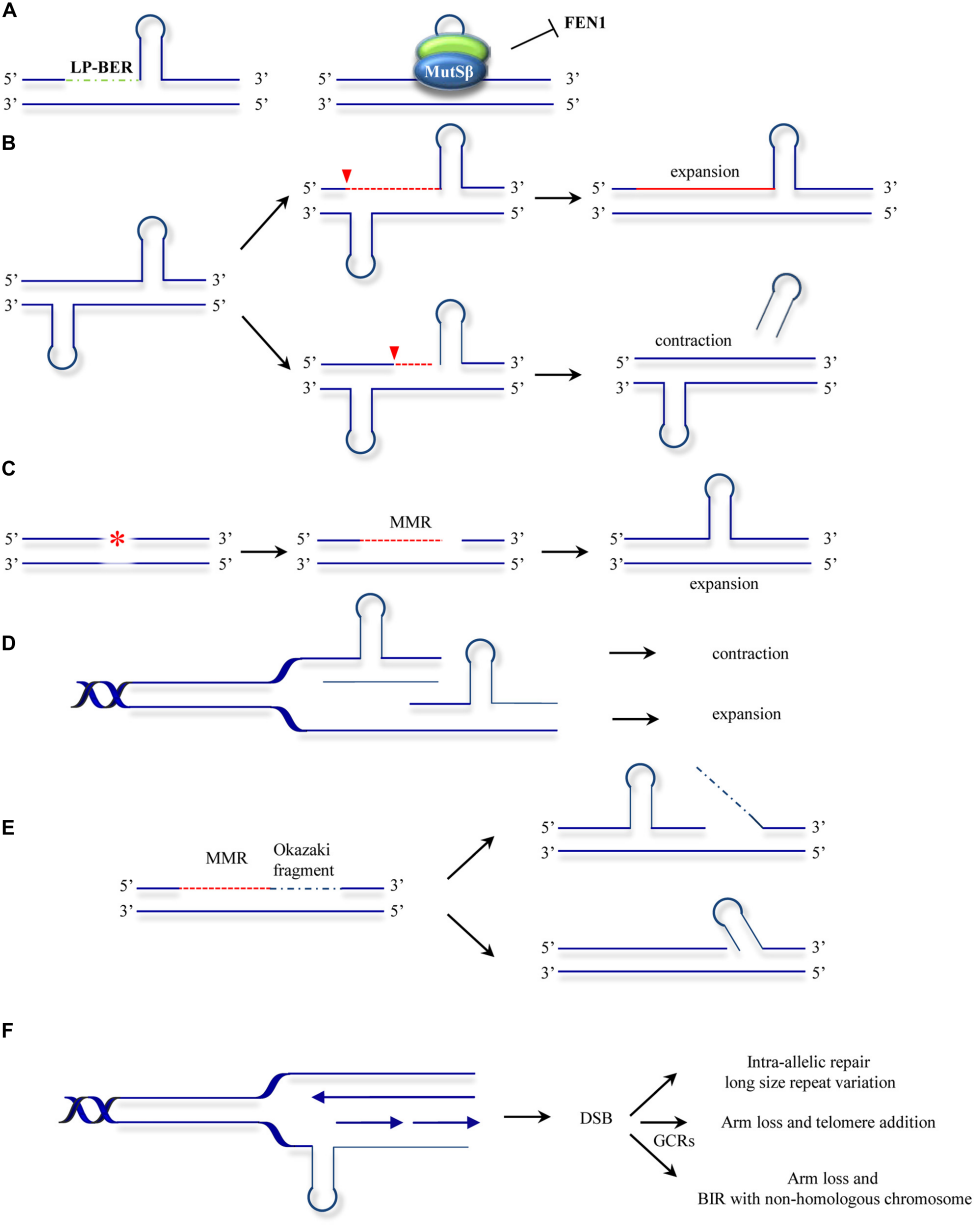
**Models for ncMMR function in repeat instability. (A)** MMR hijacks and stabilizes the hairpins formed at repeats by strand displacement during long-patch BER (LP-BER). This inhibits further processing by other repair mechanisms such as FEN1 dependent flap removal (McMurray, 2008). **(B)** Nicking in the strand opposite to a hairpin leads to unwinding of the hairpin and resynthesis across resulting in repeat expansion. Processing of the strand containing the hairpin may lead to hairpin removal and repeats contraction (adapted from Gomes-Pereira et al., 2004). **(C)** Mismatch repair processing of lesions (e.g., oxidative or alkylating damage) may lead to strand degradation and faulty resynthesis resulting in hairpin formation.** (D)** As in the hijacking model **(A)**, MutSβ stabilization of hairpins formed by polymerase slippage during replication or lack of processing of IDLs results in contraction when the hairpin is located in the template strand or expansions when the hairpin is formed in the newly synthesized strand. **(E)** Gap filling can lead to hairpin formation by strand displacement during Okazaki fragment processing (Kantartzis et al., 2012; Kim and Mirkin, 2013). **(F)** Hairpins formed at the template strand can promote MMR processing leading to DSBs. The DSBs formed can be processed by different mechanisms leading to gross chromosomal rearrangements (GCRs) or to repeat length variations (Kim et al., 2008). The models presented here are not mutually exclusive. The asterisk represents a lesion addressed by MMR. The red dotted line indicates MMR-dependent processing including strand degradation and resynthesis steps. Inverted red triangles indicated the position of EXO1 entry site. BIR, break induced replication.

The involvement of DNA repair mechanisms in repeat expansion was suggested to explain the puzzling finding that in diseases such as HD, somatic repeat instability appears most pronounced in non-proliferating tissues of the CNS (Gonitel et al., 2008) and that repeat expansion rates did not always correlate with cell division rates (Lia et al., 1998; Fortune et al., 2000; Gomes-Pereira et al., 2001). The first evidence that the MMR system contributes to repeat expansion was obtained by Manley et al. (1999). Given that a functional MMR is required for maintaining the stability of microsatellite sequences (mostly mono- and dinucleotide repeats) the authors set out to analyze whether MMR affects the stability of HD-associated CAG repeats. Surprisingly, *Msh2^-^*^/^*^-^* transgenic mice bearing a copy of the human HD exon 1 (containing the CAG repeats), showed reduced expansion of the introduced (CAG)n repeats when compared with *Msh2*^+/+^ HD exon 1 mice counterparts. Additional studies confirmed this novel mutagenic role of Msh2 in HD CAG repeat instability and HD CAG-dependent phenotypes (Kovtun and McMurray, 2001; Wheeler et al., 2003; Kovalenko et al., 2012). However, the observation that Msh2 deficiency did not completely abolish expansions suggested further hitherto unknown roles for other DNA repair processes in promoting repeat instability. Later studies provided further evidence for the non-canonical role of Msh2 in trinucleotide repeat instability, this time in (CTG)n repeat expansion associated with DM1. In contrast to the observations in HD, Msh2 absence resulted in a shift towards (CTG)n contraction rather than stabilization of the repeat size (Savouret et al., 2003). These initial findings led to a number of studies designed to decipher the role of MMR in repeat expansion. The involvement of other components of the MMR machinery was subsequently analyzed. Msh3 deficiency was found to block somatic (CTG)n expansions in DM1 knock-in mice whereas Msh6 deficiency increased the frequency of such events (Foiry et al., 2006). This suggested competition of Msh3 and Msh6 for binding to Msh2 and differential effects of MutSα and MutSβ complexes in repeat expansion (van den Broek et al., 2002). Wheeler and coworkers confirmed separate functional roles of MutSα and MutSβ complexes in HD knock-in mice and showed that whereas Msh6 protects against intergenerational contractions, Msh3 is required for CAG expansions in striatum (Dragileva et al., 2009). A model to account for the role of MutSβ in repeat instability proposes that MutSβ-dependent stabilization of secondary structures formed at the repeats and uncoupling from downstream repair events leads to instability (**Figure 3A**; McMurray, 2008). In addition, the requirement for the MutLα component PMS2 (Gomes-Pereira et al., 2004) suggested a second model where repeat instability requires a fully functional MMR (**Figure 3B**). This model is supported by the finding that Msh2-mutant mice carrying a missense mutation Msh2^G674A/G674A^ show less pronounced CTG expansions than wild type mice (Tome et al., 2009). This mutation retains mismatch recognition activity but fails to support MMR *in vitro* (Lin et al., 2004; Ollila et al., 2008; Geng et al., 2012). In an effort to gain a mechanistic insight into the MMR-dependent instability process, biochemical studies were undertaken. Using synthetic DNA substrates containing CAG or CTG slipped out structures a third model was suggested where MSH2, MSH3, and PMS2 mediate the formation of expansion intermediates prior to processing of the slip-outs (**Figure 3C**; Panigrahi et al., 2005). In this model, repair is triggered either by DNA damage in or near the TNR, or by the aberrant TNR-DNA structure itself. Subsequent excision of nucleotides is followed by error-prone repair synthesis. Despite this wealth of knowledge, the biochemical role of MutSβ in repeat instability remains controversial. MutSβ processing of CAG slip-outs *in vitro* may depend on assay conditions as well as the size, number and structure of the hairpins (Owen et al., 2005; Tian et al., 2009; Panigrahi et al., 2010; Lang et al., 2011; Zhang et al., 2012). The involvement of the MutLα heterodimer in repeat instability was also analyzed*in vitro*. Pearson and coworkers demonstrated that a functional MutLα complex is required for processing (CAG)n or (CTG)n extrusions (Panigrahi et al., 2012). How PCNA-dependent activation of MutLα endonuclease occurs in the context of non-replicating DNA was later revealed by the finding that repeat extrusions may serve as loading sites for the PCNA clamp (Pluciennik et al., 2013). These biochemical approaches have contributed to our understanding of MMR activities at unstable repeats. However, they yield only partial reactions at TNRs. Therefore, the combined use of the biochemical assays together with genetic (Dixon et al., 2004) and *in vitro* assays where complete expansion can be recapitulated (Stevens et al., 2013) may contribute further to our knowledge about the mechanisms involved in MMR-mediated instability at TNRs.

The use of other model organisms may also shed some light on the MMR mutagenic activities. Instability of TNRs was also modeled in bacteria and yeast cellular systems. In contrast to the expansion bias observed in human neurodegenerative diseases, deletion events are more frequent in bacteria and yeast (Kovtun and McMurray, 2008). In these model organisms DNA replication seems to be the major contributor to repeat instability. Several replication models for repeat expansion have been drawn on the common basis that repetitive sequences posit a challenge for replication fork progression (Kim and Mirkin, 2013). Indeed, the earliest molecular model of how repeat expansions occur was based on DNA strand slippage during replication (Kunkel, 1993). In this first model, repeats misalign during replication, resulting in formation of extrahelical DNA loops. These loops may escape from correction or become stabilized by a MutSβ-dependent mechanism. A subsequent round of replication will give rise to progeny DNA that is shorter than the template when the loop was located at the template strand or expanded when the misaligned nucleotides are in the newly synthesized strand (**Figure 3D**). Other studies suggest that MutSβ interferes with normal processing of Okazaki fragments and promotes small expansion events (**Figure 3E**; Kantartzis et al., 2012). ncMMR has also been involved in expansions via replication fork stalling, DSB formation and repair (**Figure 3F**; Kim et al., 2008). These models also have support in mammalian cell systems, as repeats have been shown to interfere with replication (Follonier et al., 2013) and the direction of replication was found to influence the frequency of expansions (Claassen and Lahue, 2007). Investigations into SV40-driven replication of plasmid templates containing (CAG)n repeats in human cells also support a role for replication in promoting repeat instability (Panigrahi et al., 2002).

Another layer of complexity is added by the potential crosstalk between different DNA repair mechanisms in repeat instability. Formation and processing of secondary structures formed at repeats suggest cooperation between MMR and other DNA repair mechanisms such as BER (Kovtun and McMurray, 2007; Kovtun et al., 2007), NER (Lin and Wilson, 2009), and chromatin modifiers (Gannon et al., 2012). Interplay of MMR with BER and NER in other cellular processes has previously been suggested (Hong et al., 2008; Schanz et al., 2009; Zhao et al., 2009; Edelbrock et al., 2013) implying that such cooperation may be a conserved feature of DNA damage response mechanisms.

Given that several of the expandable repeats associated with disease can form unusual secondary structures, and that these structures are likely to be the underlying cause of instability, it is anticipated that ncMMR plays a role in TNRs-associated diseases other than HD and DM1. In fact, Msh2 was shown to reduce intergenerational expansion of (CGG)n in a FRAXA mouse model (Lokanga et al., 2013). Analyses of (GAA)n expansions associated with FRDA though led to conflicting results (Perdomini et al., 2013). The use of alternative models such as FRDA mouse models (Bourn et al., 2012; Ezzatizadeh et al., 2012), FRDA induced pluripotent stem cells (Ku et al., 2010; Du et al., 2012) or ectopic expression of MSH2 and MSH3 in FRDA patient-derived fibroblasts (Halabi et al., 2012), may explain the discrepancies observed.

In addition, other MutS and MutL homologs may affect the stability of repeats. In this regard, a role for MutLγ in TNR expansion associated with HD has recently been described (Pinto et al., 2013). Further work is needed to clarify the potential mutagenic role of ncMMR and the MMR proteins involved in these and other repeat-associated diseases.

The models described above are not mutually exclusive and reveal a high degree of unexpected context-dependency. The mechanisms of repeat expansion may differ depending on the sequence and length of repeat, replication rates, transcription rates, chromatin state, and crosstalk between different repair mechanisms. Future work is needed to understand the relative contribution of each of these mutagenic activities to the instability of repetitive sequences.

## MMR IN THE CONTEXT OF CHROMATIN

Little is known about the influence of the chromatin context on MMR activity. Most reconstituted reactions used so far were minimal systems that cannot account for MMR as it may occur in the context of chromatin. Therefore, how the DNA packaging into chromatin affects MMR and how chromatin is restored after repair remains largely unknown. Nucleosomes inhibit MMR (Li et al., 2009) and MutSα diffusion (Gorman et al., 2010) and this barrier can be counteracted by MutSα-dependent nucleosome disassembly (Javaid et al., 2009). On the other hand, deposition of nucleosomes during replication may be tuned with MMR. By using *in vitro* modified systems containing chromatinized substrates, the groups of Jiricny and Kadyrov recently analyzed the mechanisms of nucleosome assembly during repair (Kadyrova et al., 2011; Schopf et al., 2012). These studies found coordination of MMR and nucleosome deposition initiated by the histone chaperone chromatin assembly factor 1 (CAF-1) and physical interaction between MutSα and CAF-1. CAF-1 is an essential factor in chromatin assembly in newly replicated DNA (Hoek and Stillman, 2003) and can function locally at NER sites (Green and Almouzni, 2003). The described crosstalk between MMR and CAF-1 is proposed to extend the time window available for repair by delaying chromatin assembly after replication. Histone modifications also contribute to the regulation of MMR in a chromatin context. The histone mark H3K36me3 was recently found to interact with the MMR protein MSH6 (Vermeulen et al., 2010) and facilitate MMR function by mediating its association with chromatin (Li et al., 2013). This mark is linked to actively transcribed regions but also peaks at the G1/S transition where it constitutes a chromatin signature for early replication domain boundaries (Ryba et al., 2010). This may contribute to explain the observed constitutive presence of MMR at replication factories (Lopez-Contreras et al., 2013; Sirbu et al., 2013) and its readiness for action. Importantly, mutations in SETD2, the histone methyltransferase responsible for H3K36 trimethylation, correlate with MSI found in renal cell carcinoma and Burkitt’s lymphoma cell lines that do not display genetic or epigenetic defects in MMR genes. This may provide the molecular basis for MSI in cancer with otherwise intact MMR. Similarly, histone H3 acetylation in yeast acts in concert with MMR in mutation avoidance (Kadyrova et al., 2013). These new findings pave the way for future research and a better understanding of the MMR role in disease.

## CONCLUDING REMARKS

A common theme among the DNA sequences that are subjected to mutagenic repair seems to be their tendency to present an obstacle for transcription/replication machineries to proceed. So far, only few mutation-prone genomic loci have been described, but a large fraction of the genome contains sequences with these features. Thus, it is likely that mutagenic ncMMR is not restricted to the loci described but rather influences genome integrity to a larger extend. Comprehensive studies deciphering the global finger print of mutagenic ncMMR are then needed to understand how ncMMR affects genome maintenance and contributes to disease. In addition, future studies will have to determine the factors that direct the path choice towards mutagenic or corrective activities. In the past decades the finding that MMR is involved in Lynch syndrome highlighted the relevance of this DNA repair mechanism and led to a significant progress in the field. The novel findings on the role of ncMMR in mutagenic processes and the cross-talk of MMR with other DNA repair mechanisms and with chromatin architecture are likely to renew this interest. We are confident that deeper insight into mutator and anti-mutator activities of the MMR machinery will be the basis to develop novel improved strategies for the management and treatment of MMR-associated diseases.

## Conflict of Interest Statement

The authors declare that the research was conducted in the absence of any commercial or financial relationships that could be construed as a potential conflict of interest.
